# Study on Motion Management of Pancreatic Cancer Treated by CyberKnife

**DOI:** 10.3389/fonc.2021.767832

**Published:** 2021-12-02

**Authors:** Shenghua Jing, Changchen Jiang, Xiaoqin Ji, Xiangnan Qiu, Jing Li, Xiangdong Sun, Xixu Zhu

**Affiliations:** Department of Radiation Oncology, East Region Military Command General Hospital, Nanjing, China

**Keywords:** CyberKnife, expansion margin, pancreatic cancer, SRTS, tumor motion management

## Abstract

**Purpose:**

We investigated the movement characteristics of pancreas and the clinical accuracy of tracking pancreas with the Synchrony Respiratory Tracking System (SRTS) during the CyberKnife treatment. These data provide a clinical data basis for the expansion margins of pancreatic tumor target.

**Methods and Materials:**

Forty-two patients with pancreatic cancer treated by CyberKnife were retrospectively studied. The pancreatic displacement calculated from the x-ray images collected during the time interval between two consecutive movements constituted a data set.

**Results:**

The total mean motion amplitudes and standard deviations of pancreatic tumors in SI, LR, AP, and radial directions were 3.66 ± 1.71 mm, 0.97 ± 0.62 mm, 1.52 ± 1.02 mm, and 1.36 ± 0.49 mm, respectively. The overall mean correlation errors and standard deviations were 0.82 ± 0.46 mm, 0.47 ± 0.33 mm, 0.41 ± 0.24 mm, and 0.98 ± 0.37 mm, respectively. The overall mean prediction errors and standard deviations were 0.57 ± 0.14 mm, 0.62 ± 0.28 mm, 0.39 ± 0.17 mm, and 1.58 ± 0.36 mm, respectively. The correlation errors and prediction errors of pancreatic tumors at different anatomical positions in SI, LR, and AP directions were statistically significant (*p* < 0.05).

**Conclusions:**

The tumor motion amplitude, the tumor location, and the treatment time are the main factors affecting the tracking accuracy. The pancreatic tumors at different anatomical locations should be treated differently to ensure sufficient dose coverage of the pancreatic target area.

## Introduction

Pancreatic cancer is one of the most aggressive tumors, and there is almost no effective treatment method at present. Even in resected patients, the prognosis is still very poor, and the incidence of local recurrence (1) is between 20% and 60%. Stereotactic radiosurgery for pancreatic cancer has shown promising early results (2). SBRT can maximize the protection of surrounding normal tissue by forming a significant dose gradient around the prescription dose (3). Current evidence suggests that increasing the dose of SBRT may further improve patient outcomes (4). However, the increase of dose is limited by toxicity of surrounding normal organs. During SBRT treatment of pancreas, the surrounding normal organs, stomach, and duodenum (5) are highly sensitive to radiation and adjacent to the pancreas. Due to breathing, digestion, and heartbeat, the boundary between the tumor and nearby organs is blurred. This internal target movement may lead to insufficient local dose of tumor and excessive dose of normal organs at risk (OARs) (6).

In order to reduce the adverse effects of internal organ movement in the treatment, and compensate for the unquantified geometric uncertainty in target tumor location, scholars usually apply general margins to clinical target volume (CTV) to the planning target volume (PTV) margins. This margin estimation may not include the “current” range of motion presented by the pancreas (7, 8). At present, different methods have been proposed to deal with respiratory movement (9), such as the abdominal compression technique (10), respiratory gating technique (11), breath holding technique (12), internal-target-volume (ITV) (13), and simultaneous dynamic tumor tracking (DTT) technique.

Tumor tracking is an advanced method to manage respiratory movement. This method reduces the size of PTV. This can improve targeting and achieve better tumor control, and minimize radiation to normal tissues (14). However, there is little clinical guidance on the management of pancreatic cancer patients.

In this paper, we analyzed 219 data sets recorded by 42 patients with pancreatic cancer. By tracking external markers and implanted fiducials through the stereo x-ray imaging, we monitored the movement data of pancreas during the treatment to quantify the movement of pancreas, and deeply studied the characteristics of fractional internal movement of pancreas. This study aims to answer three key clinical questions: (1) motion characteristics of pancreatic tumors under free breathing; (2) the accuracy and related factors of tracking pancreas by the CyberKnife SRT system; and (3) the expansion margin of the pancreatic target is guided by the movement characteristics of pancreas and the tracking accuracy of the SRT system.

## Methods and Materials

### Data Source

From January 2017 to December 2020, 42 patients with locally advanced pancreatic cancer received CyberKnife radiotherapy using SRTS in the radiotherapy department. The treatment characteristics of patients are listed in **Supplementary Table 1**. Previous research on the number of implanted fiducials showed that less than three fiducials can only calculate the three-dimensional translation deviation, but not the rotation angle deviation. The correlation models established with three or more fiducials are more stable compared with those less than three fiducials. In this study, the pancreas was divided into pancreatic head, neck, body, and tail to analyze the motion management of pancreatic cancer. It was necessary to ensure that the fiducials were in their respective anatomical positions to avoid overlapping and affecting the results. Finally, before treatment, two to five fiducials were implanted into or around the tumor under the guidance of CT or ultrasonic endoscopy. The patient was fixed with a vacuum pad, and his/her arm was placed above the head. Half an hour before CT positioning, the oral contrast agent was taken. During CT positioning, the contrast agent was injected intravenously to obtain the CT sectional image at the end of inspiration, with a layer thickness of 1 mm. The patient breathed freely throughout the treatment. There was no respiratory training for the patient before CT positioning and CyberKnife treatment. The design of the treatment plan was based on end-inspiratory CT image, and PTV was based on the expansion of GTV by 4 mm in all directions. The dose of PTV was defined as an isodose line of 65% to 76%, where 100% was normalized to the maximum dose.

The CyberKnife synchrony tracking system will continuously synchronize the beam transmission and breathing, thereby tracking the tumor targets without interrupting the treatment or moving the patient. After each treatment, the CyberKnife system will save a log file containing the centroid displacement of fiducials in the superior–inferior (SI), left–right (LR), and anterior–posterior (AP) directions. This can be used to analyze the organ movement during the beam transmission. The pancreatic movement is defined as the centroid displacement of fiducials relative to the planned position. Some fiducials migrate or rotate during treatment. If the fiducials exceeded the respective anatomical range, the data sets of the subsequent treatment were discarded. The respiratory motion data, pancreatic motion data, correlation error data and prediction error data were extracted from the treatment log files.

### Respiratory Movement Data

Three optical markers were used to record external respiratory signals in real time. These markers were optical fiber terminals for transmitting LED signals (15, 16). Before treatment, three infrared markers were pasted on the patient’s chest or abdomen. In this study, the patients were treated with two or three external optical markers. The 3D position of external markers was continuously measured by the stereo camera system at a frequency of about 30 Hz. The distance of each marker along the main axis of movement was recorded for the correlation model.

### Baseline Drift

It is reported that the external substitution movement is closely related to the internal tissue movement (17). However, this correlation may change due to a baseline drift of the patient’s breathing and gradual relaxation of muscles. Baseline drift was defined as the slow changes of the respiratory baseline in one direction overtime (18). Baseline drift was calculated by subtracting the absolute value of the lowest point from the highest point of the baseline, and then dividing by time. Malinowski (19) investigated patients with lung and pancreatic tumors, and the relationship between substitutes and tumor location changed in 63% of cases.

### Correlation Model

A pair of orthogonal x-ray tubes was used to take many x-ray images of patients. At different stages of the respiratory cycle, the position and direction of several fiducials implanted in or near the tumor were monitored until the SRT system showed that the respiratory cycle was 100% covered. The position of fiducials was automatically extracted from x-ray images, and its three-dimensional coordinates were reconstructed in the patient coordinate system through back projection. Finally, the marker configuration was registered to the marker configuration in the planned CT scanning images to determine the location of the tumor.

Therefore, the average errors of two or three independent models coupled with the external marker were used for each component of the movement. We chose to use the average value, because the output of the correlation model transmitted to the robot controller was the average value of all the external markers.

### Prediction Model

Another component of the SRT system is the prediction model. The tumor location information was obtained 115 ms in advance in the SRT system through the prediction model. The tumor identification and beam adjustment were completed within 115 ms through the SRT system (20).

The prediction error was calculated by comparing the predicted location with the actual location after 115 ms. The overall mean errors and the standard deviations of each fraction were calculated for each patient in the SI, LR, and AP directions. The Modeler.log, the Predictor.log, the ModelPoints.log, the Markers.log, and the ERsiData.log were in the log files (21).

### Data Analysis for Correlation and Prediction Errors

Treatment may be interrupted by excessive coughing, deep breathing, and slight displacement. In these cases, all existing data points were deleted by resetting the model, and a new correlation model was constructed. The output of the correlation model was used to calculate the amplitude of tumor movement. The amplitude was calculated by using a movement range of 5% to 95%. Only data matched with the dose delivery of the treatment in time were used for analysis.

The predictor provided an estimate of the future target position using the past movement pattern. The output of the correlation model for each direction component was predicted separately. The prediction error was calculated by comparing the predicted location with the actual location after 115 ms. Similarly, only data that matched with the dose delivery of the treatment in time was used for analysis. The maximum prediction error, mean prediction error, and standard deviations of each treatment were calculated. The overall mean errors and the standard deviations of each fraction were calculated for each patient in the SI, LR, and AP directions. The radial error was calculated by summing the square roots in each direction.

### Statistical Analysis

Data of each patient were calculated and expressed as overall mean ± standard deviation. The Pearson correlation coefficient *r* was evaluated with an uncorrelated test. Comparisons were performed using *t*-test, and the differences were considered significant when *p*-value was less than 0.05. The statistical analyses are based on SPSS statistics of IBM.

The factors for the tracking accuracy were estimated through the multivariate regression analysis. Correlation errors, prediction errors, and radial errors were specified as dependent variables. Seven parameters, namely, baseline drift, respiratory amplitude, respiratory cycle, treatment time, tumor volume, tumor motion amplitude and tumor anatomical location, were extracted as independent variables.

## Results

### Tumor Movement Characteristics

The average duration of each data set was 45.9 min, and the average volume of tracking tumor was 11.7 ± 15.3 cm^3^. In **Supplementary Table 2**, the overall mean and standard deviation of tumor motion amplitude in SI, LR, AP, and radial directions were 3.65 ± 1.71 mm, 0.97 ± 0.62 mm, 1.52 ± 1.02 mm, and 1.36 ± 0.49 mm, respectively. The overall mean and standard deviation of respiratory amplitude were 21.49 ± 17.05 mm, 5.01 ± 4.99 mm, 6.18 ± 9.57 mm, and 7.62 ± 2.43 mm, respectively. The respiratory amplitude and tumor motion amplitude in SI direction were significantly greater than that in the LR and AP directions (*p* = 0.000).

### Tumor Movement Characteristics at Different Anatomical Positions

The centroid movement of fiducials was used as an alternative to pancreatic movement. The value was continuously recorded over time, and 219 data sets were analyzed. The overall mean and standard deviations of tumor motion amplitude in SI, LR, AP, and radial directions are as follows (**Table 1**): (1) pancreatic head: 3.16 ± 1.38 mm, 1.14 ± 0.59 mm, 1.66 ± 0.74 mm, and 1.24 ± 0.29 mm; (2) pancreatic neck: 3.72 ± 0.81 mm, 0.88 ± 0.59 mm, 1.02 ± 0.31 mm, and 1.13 ± 0.20 mm; (3) pancreatic body: 3.85 ± 1.80 mm, 0.92 ± 0.70 mm, 1.42 ± 1.18 mm, and 1.41 ± 0.60 mm; and (4) pancreatic tail: 3.74 ± 2.10 mm, 0.78 ± 0.43 mm, 1.70 ± 1.04 mm, and 1.45 ± 0.42 mm.

**Table 1:** Overview of pancreatic tumor motion at different anatomical locations.

Locations and directions	Mean (mm)	SD (mm)	Range (mm)
Pancreatic head (*N* = 44)			
SI	3.16	1.38	1.25–5.79
LR	1.14	0.59	0.45–2.54
AP	1.66	0.74	0.59–3.14
Radial	1.24	0.29	0.79–1.84
Pancreatic neck (*N* = 40)			
SI	3.72	0.81	1.29–4.20
LR	0.88	0.59	0.26–1.84
AP	1.02	0.31	0.54–1.64
Radial	1.13	0.20	0.83–1.34
Pancreatic body (*N* = 62)			
SI	3.85	1.80	1.43–11.39
LR	0.92	0.70	0.17–3.08
AP	1.42	1.18	0.22–5.48
Radial	1.41	0.60	0.64–4.18
Pancreatic tail (*N* = 73)			
SI	3.74	2.10	1.99–9.76
LR	0.78	0.43	0.25–1.62
AP	1.70	1.04	0.58–4.57
Radial	1.45	0.42	0.85–2.18

### Correlation and Prediction Errors

**Figure 1:**
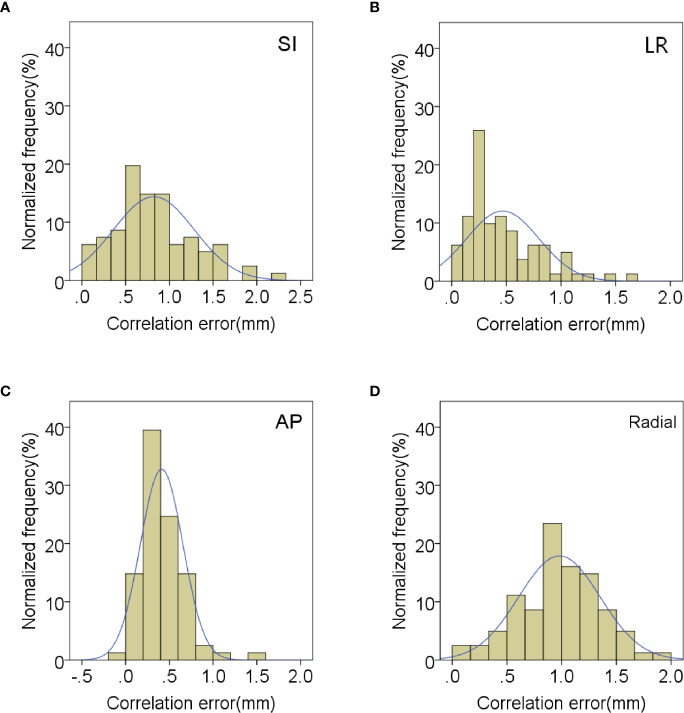
Distribution of mean correlation errors in **(A)** SI, **(B)** LR, **(C)** AP and **(D)** radial directions.

**Figure 2:**
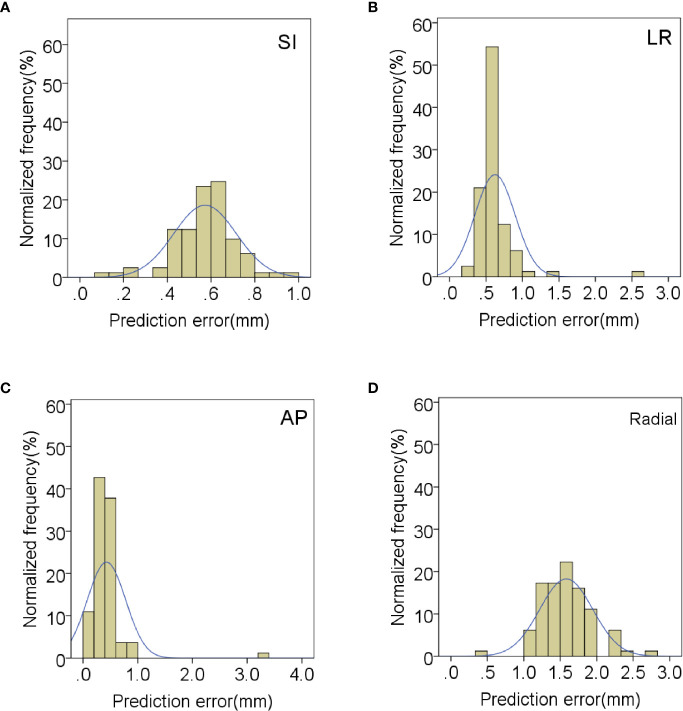
Distribution of mean prediction errors in **(A)** SI, **(B)** LR, **(C)** AP and **(D)** radial directions.

In order to evaluate the correlation and prediction errors in clinical log files, 219 data sets of 42 patients were analyzed. The histograms of correlation and prediction errors in all directions are shown in **Figures 1**, **2**, respectively. The overall mean correlation and prediction errors at different anatomical locations are summarized in **Table 2**. The average correlation and prediction errors in SI, LR, and AP directions were very small, and the average correlation error in the radial direction was less than 1 mm. The correlation errors of tumors located in pancreatic neck in SI, LR, and AP directions were significantly greater than that in other parts (SI direction: 1.02 ± 0.52 mm vs. 0.84 ± 0.31 mm, 0.83 ± 0.34 mm, and 0.59 ± 0.26 mm; LR direction: 0.57 ± 0.36 mm vs. 0.56 ± 0.20 mm, 0.45 ± 0.25 mm, and 0.23 ± 0.13 mm; AP direction: 0.62 ± 0.27 mm vs. 0.48 ± 0.21 mm, 0.38 ± 0.15 mm, and 0.32 ± 0.17 mm, *p* < 0.05). The prediction errors in SI and AP directions were gradually increased from pancreatic head to pancreatic tail (SI direction: 0.49 ± 0.11 mm vs. 0.54 ± 0.13 mm, 0.58 ± 0.14 mm, and 0.62 ± 0.17 mm; AP direction: 0.35 ± 0.08 mm vs. 0.39 ± 0.11 mm, 0.42 ± 0.14 mm, and 0.43 ± 0.15 mm, *p* < 0.05).

**Table 2:** Summary of the correlation and prediction errors in 219 fractions.

Locations and directions (Data sets)		Mean (mm)		SD (mm)		Range (mm)	
		Correlation error	Prediction error	Correlation error	Prediction error	Correlation error	Prediction error
Pancreatic head (*N* = 44)	SI	0.84	0.49	0.31	0.11	0.25–4.01	0.11–3.03
	LR	0.56	0.51	0.20	0.10	0.13–2.26	0.28–4.08
	AP	0.48	0.35	0.21	0.08	0.20–2.02	0.1–3.99
	Radial	1.01	1.55	0.36	0.20	0.04–3.39	1.08–7.49
Pancreatic neck (*N* = 40)	SI	1.02	0.54	0.52	0.13	0.17–4.58	0.21–4.51
	LR	0.57	0.61	0.36	0.09	0.08–3.23	0.44–3.91
	AP	0.62	0.39	0.27	0.11	0.12–2.43	0.06–1.38
	Radial	0.95	1.27	0.35	0.13	0.08–3.61	0.34–9.32
Pancreatic body (*N* = 62)	SI	0.83	0.58	0.34	0.14	0.06–3.98	0.26–3.10
	LR	0.45	0.66	0.25	0.19	0.02–2.15	0.36–3.21
	AP	0.38	0.42	0.15	0.14	0.08–1.82	0.13–2.05
	Radial	0.96	1.62	0.41	0.39	0.15–3.26	0.35–7.94
Pancreatic tail (*N* = 73)	SI	0.59	0.62	0.26	0.17	0.15–2.60	0.40–3.41
	LR	0.23	0.71	0.13	0.54	0.06–1.27	0.45–3.39
	AP	0.32	0.43	0.17	0.15	0.11–1.88	0.27–2.59
	Radial	0.89	1.64	0.27	0.48	0.45–2.92	1.14–7.95
Total (*N* = 219)	SI	0.82	0.57	0.46	0.14	0.06–4.58	0.11–4.51
	LR	0.47	0.62	0.33	0.28	0.02–3.23	0.28–4.08
	AP	0.41	0.39	0.24	0.17	0.08–2.43	0.06–3.99
	Radial	0.98	1.58	0.37	0.36	0.04–3.61	0.35–9.51

For the anatomical location of pancreatic tumors, the correlation errors and prediction errors of different anatomical locations in SI, LR, and AP directions were statistically significant (correlation errors: *p* = 0.006, 0.00, and 0.038, respectively; prediction errors: *p* = 0.011, 0.048, and 0.031, respectively). However, the correlation errors and prediction errors of pancreatic tumors in radial direction at different anatomical locations were not statistically significant (*p* = 0.401 and 0.196).

### Correlations of Tracking Parameters

The correlations of tracking parameters were counted to determine their influence on the correlation errors and prediction errors. The influencing factors include individual patient differences (respiratory cycle and respiratory amplitude), tumor anatomical location, tumor movement amplitude, baseline drift, tumor volume, and treatment time. The Pearson correlation was used to analyze the correlation between seven factors and errors. The results of correlation analysis were summarized in **Supplementary Table 3**. The correlation errors and prediction errors in all directions were significantly less correlated with tumor motion amplitude (*r* > 0.3, *p* < 0.01). The correlation errors in the LR direction and the prediction errors in the AP and radial directions were correlated with tumor motion amplitude (*r* > 0.5, *p* < 0.01). The correlation errors in all directions were significantly less correlated with treatment time (*r* > 0.3, *p* < 0.01). The correlation errors in SI, LR, and AP directions were significantly less correlated with tumor anatomical location (*r* > 0.3, *p* < 0.01). The correlation error in the LR direction was correlated with tumor anatomical location (*r* > 0.5, *p* < 0.01). The correlation error in the SI direction was significantly less correlated with baseline drift (*r* = −0.3, *p* = 0.006). The correlation error in the AP direction was significantly less correlated with tumor volume (*r* = 0.332, *p* = 0.002).

**Figure 3:**
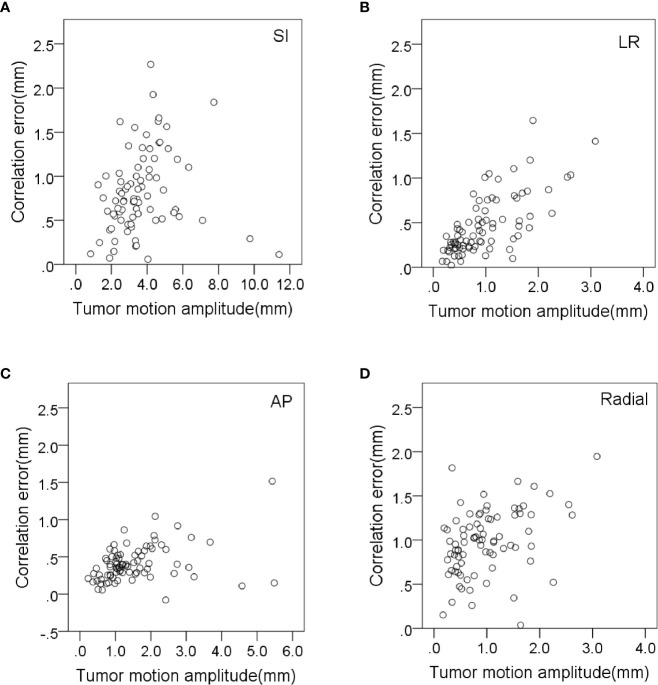
Variation of correlation errors with tumor motion amplitude in **(A)** SI, **(B)** LR, **(C)** AP and **(D)** radial directions.

**Figure 4:**
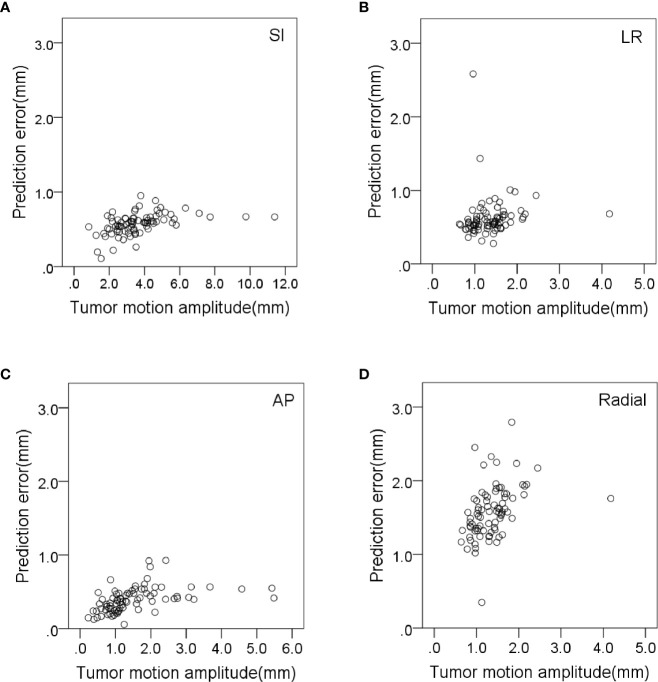
Variation of prediction errors with tumor motion amplitude in **(A)** SI, **(B)** LR, **(C)** AP and **(D)** radial directions.

The prediction error in the AP direction was significantly less correlated with respiratory amplitude and tumor volume (*r* = 0.418 and 0.385, *p* < 0.01). The prediction error in radial direction was significantly less correlated with tumor volume and respiratory cycle (*r* = 0.438 and −0.317, *p* < 0.01). The prediction error in the LR direction was significantly less correlated with baseline drift and respiratory rate (*r* = 0.336 and −0.446, respectively, *p* < 0.01). The variation of correlation error and prediction error with tumor motion amplitude in all directions is shown in **Figures 3**, **4**, respectively. Other parameters had no significant correlation with correlation or prediction error.

### Tumor Expansion Margins

There are three factors for the boundary expansion during the treatment with the CyberKnife SRT system: (I) the aiming accuracy of CyberKnife; (II) correlation error; and (III) prediction error. Previous studies have found that the mechanical error was 0.1 mm and the maximum position uncertainty was 0.3 mm. In the monthly quality assurance program of CyberKnife in Indianapolis (22), the aiming error is 0.5 mm.

In this paper, the correlation errors and prediction errors were correlated with the tumor motion amplitude. In our analysis, the correlation errors were extracted from the model points log file. The mean, minimum, maximum, and standard deviations of the correlation errors and prediction errors of each course were measured. Two standard deviations from the mean value of each anatomical direction were used to ensure 95% coverage of modeling points. Similarly, three standard deviations from the mean value of each anatomical direction were used to ensure 99% coverage of modeling points. However, if the minimum or maximum deviation was less than two standard deviations, they were replaced. The prediction errors in SI, LR, and AP directions did not contain direction information, so the prediction error in radial direction will be greater than the actual value. Therefore, the correlation and prediction errors in the radial direction are not shown in **Table 3**.

**Table 3:** Statistics of correlation, prediction, and total errors of pancreatic tumors at different anatomical locations.

Locations and directions		Correlation error (mm)		Prediction error (mm)		Aiming accuracy (mm)	Total error (mm)	
		95% CI	99% CI	95% CI	99% CI		95% CI	99% CI
Pancreatic head	SI	1.46	1.77	0.83	1	0.5	2.79	3.27
	LR	0.97	1.17	0.71	0.81	0.5	2.18	2.48
	AP	0.9	1.11	0.51	0.59	0.5	1.91	2.2
Pancreatic neck	SI	2.49	3.03	0.62	0.66	0.5	3.61	4.19
	LR	1.59	1.95	0.63	0.68	0.5	2.72	3.13
	AP	1.16	1.43	0.61	0.72	0.5	2.27	2.65
Pancreatic body	SI	1.87	2.39	0.82	0.94	0.5	3.19	3.83
	LR	0.95	1.2	1.04	1.23	0.5	2.49	2.93
	AP	0.68	0.83	0.7	0.84	0.5	1.88	2.17
Pancreatic tail	SI	1.11	1.37	0.9	1.04	0.5	2.51	2.91
	LR	0.49	0.62	1.79	2.33	0.5	2.78	3.45
	AP	0.66	0.83	0.73	0.88	0.5	1.89	2.21

## Discussion

The results of a study on 4DCT of pancreatic tumors by Sarkar et al. showed the daily breathing inconsistency in the pancreas SBRT (23). This further indicated that the isotropic ITV edge expansion may not be appropriate because it cannot be fully considered the movement of inter- and intra-fractions. This paper analyzed the movement characteristics of pancreatic tumors at different anatomical positions through clinical log files and successfully described the correlation errors, prediction errors, and overall error of the synchronous tumor tracking system. This paper provides appropriate information for the expansion of clinical target GTV.

Tumor motion amplitude was significantly correlated with correlation errors and prediction errors (**Figures 3**, **4**). This indicates that tumors with greater movement amplitude may produce greater tracking errors. Winter et al. studied the relationship between the tracking errors and tumor motion amplitude in patients with liver cancer. They showed that there was a strong correlation between prediction error and target amplitude (24). They also reported that the correlation error was related to the target tumor volume. Our data showed that the correlation error and prediction error in AP direction were less correlated with target tumor volume (*r* = −0.332 and 0.385, *p* < 0.01).

It is reported that the large respiratory motion amplitude of tumors is related to the baseline drift. This will affect the reproducibility of the position between tumors (25). This correlation will have a significant impact on the calculation of ITV and PTV margins. However, this is inconsistent with our findings. Our results showed that the tumor motion amplitude was not correlated with baseline drift in all directions (**Supplementary Figure 1**).

There are some limitations in this paper. First, tumor motion amplitude was from the movement data of fiducials. Although the use of implanted fiducials in abdominal tumor treatment has increased significantly, some potential problems related to their use need to be further studied. For example, the distance and spatial relationship between multiple implant fiducials and tumors may change because of the treatment and/or disease-related organ swelling or contraction. In addition, due to the differential movement caused by organ deformation, the distance between fiducials and the tumor may change during the respiratory cycle (26).

Second, the relationship among errors and prediction models and motion is based on external LED markers. The correlation and prediction models were constructed based on external LED signals through the SRT system. Therefore, the location of LED may affect the model errors. However, it is difficult to extract specific parameters from each patient’s LED marker data, because CyberKnife treatment lasted longer than IMRT. In addition, each patient in this study can breathe freely during CyberKnife treatment. Although these abnormal data have been excluded from statistics, there were some irregular breathing patterns in the respiratory data.

Previous studies on lung patients treated with CyberKnife synchronous tracking system showed that correlation errors were not correlated with the amplitude and variability of LED markers. This indicated that the respiratory model was not the main factor for the tracking accuracy. Our study on pancreatic patients treated with CyberKnife synchronous tracking system shows that correlation errors were not correlated with the amplitude and variability of LED markers in all directions. However, only the prediction errors in AP direction were correlated with the amplitude and variability of LED markers. Our results show that the motion amplitude and location of pancreatic tumors are the main factors for the tracking accuracy. The tumor movement is mainly caused by the patient’s breathing. Therefore, it is urgent to determine the effects of breathing mode on the tracking accuracy of different tumors in future clinical studies.

## Conclusions

The inter-fraction movement of pancreas has been considered as one of the main limiting factors for the increase of pancreatic dose during the pancreatic cancer radiotherapy for a long time (27). A detailed understanding of pancreatic movement helps to understand the nature and extent of the adverse effects of uncertainty. In this study, we studied the internal motions and the tracking accuracies of 42 patients with pancreatic cancer treated with CyberKnife and analyzed the tracking accuracies of different anatomical locations. The results show that the tumor motion amplitude, the anatomical location of tumor, and the treatment time were the main factors for the tracking accuracy. The results emphasize the importance of the anatomical location of pancreatic tumors to the expansion margins. The pancreatic tumors at different anatomical locations should be treated differently in the calculation of the expansion margins, because of the amplitude and randomness of pancreatic movement. This is important in future pancreatic radiotherapy to ensure adequate dose coverage of pancreatic targets.

It should be noted that the CT scan images of the treatment plan in this study is based on the end of inspiration rather than the end of expiration. There are many studies on the difference between the end exhale and the end inhale position, and it is true that the end exhale position has many advantages. The end exhale position was the most stable position in the breathing cycle and tumors spent more time closer to the end exhale position than to the end inhale position. We found more overlapping volume of duodenum and stomach at the end inhale position compared to that at the end exhale position in pancreatic cancer with 4DCT scanning. Therefore, a dose to the duodenum was higher when treating during the inspiratory phase than during the expiratory phase. In order to understand the results of this study, we need to distinguish the differences between these two methods.

## Data Availability Statement

The original contributions presented in the study are included in the article/**Supplementary Material**. Further inquiries can be directed to the corresponding authors.

## Author Contributions

SJ and XJ designed the study and wrote the manuscript. SJ, XJ, and JL specially collected clinical data. CJ and XQ used statistics to analyze and integrate research data. Thanks to XS for supervising the manuscript research. Thanks to XZ for the feasibility analysis of the research conclusions and other data. All authors contributed to the article and approved the submitted version.

## Conflict of Interest

The authors declare that the research was conducted in the absence of any commercial or financial relationships that could be construed as a potential conflict of interest.

## Publisher’s Note

All claims expressed in this article are solely those of the authors and do not necessarily represent those of their affiliated organizations, or those of the publisher, the editors and the reviewers. Any product that may be evaluated in this article, or claim that may be made by its manufacturer, is not guaranteed or endorsed by the publisher.

## References

[1] Dagoglu N Callery M Moser J Tseng J Kent T Bullock A. Stereotactic Body Radiotherapy (SBRT) Reirradiation for Recurrent Pancreas Cancer. J Cancer (2016) 7(3):283–8. doi: 10.7150/jca.13295 PMC4747882 26918041

[2] Chuong MD Springett GM Freilich JM Park CK Weber JM Mellon EA. Stereotactic Body Radiation Therapy for Locally Advanced and Borderline Resectable Pancreatic Cancer Is Effective and Well Tolerated. Int J Radiat Oncol Biol Phys (2013) 86:516–22. doi: 10.1016/j.ijrobp.2013.02.022 23562768

[3] Ding Y Campbell WG Miften M Vinogradskiy Y Jones BL. Quantifying Allowable Motion to Achieve Safe Dose Escalation in Pancreatic SBRT. Pract Radiat Oncol (2019) 9(4):432–442. doi: 10.1016/j.prro.2019.03.006 PMC6592725 30951868

[4] Golden EB Chhabra A Chachoua A Adams S Donach M Fenton-Kerimian M. Local Radiotherapy and Granulocyte-Macrophage Colony-Stimulating Factor to Generate Abscopal Responses in Patients With Metastatic Solid Tumours: A Proof-of-Principle Trial. Lancet Oncol (2015) 16:795–803. doi: 10.1016/S1470-2045(15)00054-6 26095785

[5] Yang W Fraass BA Reznik R Nissen L Lo S Jamil LH. Adequacy of Inhale/Exhale Breathhold CT Based ITV Margins and Image-Guided Registration for Free-Breathing Pancreas and Liver SBRT. Radiat Oncol (London England) (2014) 9:11. doi: 10.1186/1748-717X-9-11 PMC3896695 24401365

[6] Ding Y Barrett HH Kupinski MA Vinogradskiy Y Miften M Jones BL. Objective Assessment of the Effects of Tumor Motion in Radiation Therapy. Med Phys (2019) 46(7):3311–23. doi: 10.1002/mp.13601 PMC6625917 31111961

[7] Cao Y Zhu X Ju X Liu Y Yu C Sun Y. Optimization of Dose Distributions of Target Volumes and Organs at Risk During Stereotactic Body Radiation Therapy for Pancreatic Cancer With Dose-Limiting Auto-Shells. Radiat Oncol (2018) 13(1):1–6. doi: 10.1186/s13014-018-0956-7 29357875 PMC5778643

[8] Karava K Ehrbar S Riesterer O Roesch J Glatz S Klöck S. Potential Dosimetric Benefits of Adaptive Tumor Tracking Over the Internal Target Volume Concept for Stereotactic Body Radiation Therapy of Pancreatic Cancer. Radiat Oncol (2017) 12(1):175. doi: 10.1186/s13014-017-0906-9 29121945 PMC5680781

[9] Wilke Lotte Andratschke Nicolaus Blanck Oliver. ICRU Report 91 on Prescribing, Recording, and Reporting of Stereotactic Treatments with Small Photon Beams : Statement from the DEGRO/DGMP Working Group Stereotactic Radiotherapy and Radiosurgery. Strahlenther Onkol (2019) 195(3):193–8. doi: 10.1007/s00066-018-1416-x 30649567

[10] Murray B Forster K Timmerman R. Frame-Based Immobilization and Targeting for Stereotactic Body Radiation Therapy. Med Dosim (2007) 32:86–91.13. doi: 10.1016/j.meddos.2007.01.005 17472887

[11] Taniguchi CM Murphy JD Eclov N Atwood TF Kielar KN Christman-Skieller C. Dosimetric Analysis of Organs at Risk During Expiratory Gating in Stereotactic Body Radiation Therapy for Pancreatic Cancer. Int J Radiat Oncol Biol Phys (2013) 85:1090–5. doi: 10.1016/j.ijrobp.2012.07.2366 23273994

[12] Dawson LA Brock KK Kazanjian S Fitch D McGinn CJ Lawrence TS. The Reproducibility of Organ Position Using Active Breathing Control (ABC) During Liver Radiotherapy. Int J Radiat Oncol Biol Phys (2001) 51:1410–21. doi: 10.1016/S0360-3016(01)02653-0 11728702

[13] Ehrbar S Jöhl A Tartas A Stark LS Riesterer O Klöck S. Itv, Midventilation, Gating or Couch Tracking - A Comparison of Respiratory Motion-Management Techniques Based on 4D Dose Calculations. Radiother Oncol (2017) 124:80–8. doi: 10.1016/j.radonc.2017.05.016 28587761

[14] Papalazarou C Klop GJ Milder MTW Marijnissen JPA Gupta V Heijmen BJM. CyberKnife With Integrated CT-On-Rails: System Description and First Clinical Application for Pancreas SBRT. Med Phys (2017) 44:4816–27. doi: 10.1002/mp.12432 28657157

[15] Inoue M Okawa K Taguchi J Hirota Y Ohta S. Factors Affecting the Accuracy of Respiratory Tracking of the Image-Guided Robotic Radiosurgery System. Japanese J Radiol (2019) 37(10):727–34. doi: 10.1007/s11604-019-00859-7 31367890

[16] Ferris WS Kissick MW Bayouth JE Culberson WS Smilowitz JB. Evaluation of Radixact Motion Synchrony for 3D Respiratory Motion: Modeling Accuracy and Dosimetric Fidelity. J Appl Clin Med Phys (2020) 21(9):96–106. doi: 10.1002/acm2.12978 PMC7497925 32691973

[17] Zhang H Zhao G David D Yaoqin X. Determination of Acquisition Frequency for Intrafractional Motion of Pancreas in CyberKnife Radiotherapy. Sci World J (2014) 2014:408019. (2014-5-13). doi: 10.1155/2014/408019 PMC4053084 24959616

[18] Akino Y Shiomi H Sumida I Isohashi F Seo Y Suzuki O. Impacts of Respiratory Phase Shifts on Motion Tracking Accuracy of the CyberKnife Synchrony Respiratory Tracking System. Med Phys (2019) 46(9):3757–66. doi: 10.1002/mp.13523 30943311

[19] Malinowski K McAvoy TJ George R Dietrich S D’Souza WD. Incidence of Changes in Respiration-Induced Tumor Motion and Its Relationship With Respiratory Surrogates During Individual Treatment Fractions. Int J Radiat Oncol Biol Phys (2012) 82:1665–73. doi: 10.1016/j.ijrobp.2011.02.048 21498009

[20] Subedi G Karasick T Grimm J Jain S Xue J Xu Q. Factors That May Determine the Targeting Accuracy of Image-Guided Radiosurgery. Med Phys (2015) 42(10):6004–10. doi: 10.1118/1.4930961 26429275

[21] Hoogeman M Prévost J-B Nuyttens J P?Ll J Levendag P Heijmen B. Clinical Accuracy of the Respiratory Tumor Tracking System of the CyberKnife: Assessment by Analysis of Log Files. Int J Radiat Oncol Biol Phys (2009) 74(1):297–303. doi: 10.1016/j.ijrobp.2008.12.041 19362249

[22] Pepin EW Wu H Zhang Y Lord B. Correlation and Prediction Uncertainties in the CyberKnife Synchrony Respiratory Tracking System. Med Phys (2011) 38(7):4036–44. doi: 10.1118/1.3596527 PMC3139505 21859002

[23] Sarkar V Lloyd S Paxton A Huang L Su FC Tao R. Daily Breathing Inconsistency in Pancreas SBRT: A 4DCT Study. J Gastrointestinal Oncol (2018) 9(6):989–95. doi: 10.21037/jgo.2018.09.08 PMC6286933 30603117

[24] Winter JD Wong R Swaminath A Chow T. Accuracy of Robotic radiosurgical Liver Treatment Throughout the Respiratory Cycle. Int JRadiat Oncol Biol Phys (2015) 93:916–24. doi: 10.1016/j.ijrobp.2015.08.031 26530762

[25] Chan MK Kwong DL Tam E Tong A Ng SCY. Quantifying Variability of Intrafractional Target Motion in Stereotactic Body Radiotherapy for Lung Cancers. J Appl Clin Med Phys (2013) 14(5):140–52. doi: 10.1120/jacmp.v14i5.4319 PMC5714563 24036866

[26] Pettersson N Oderinde OM Murphy J Simpson D Cervio LI. Intrafractional Relationship Changes Between an External Breathing Signal and Fiducial Marker Positions in Pancreatic Cancer Patients. J Appl Clin Med Phys (2020) 21:153–61. doi: 10.1002/acm2.12841 PMC7075406 32170900

[27] Casamassima F Cavedon C Francescon P Stancanello J Avanzo M Cora S. Use of Motion Tracking in Stereotactic Body Radiotherapy: Evaluation of Uncertainty in Off-Target Dose Distribution and Optimization Strategies. Acta Oncol (2016) 45(7):943–7. doi: 10.1080/02841860600908962 16982561

