# Supplementary figures and images for: Study on Motion Management of Pancreatic Cancer Treated by CyberKnife
Source: Front Oncol. 2021 Dec 2;11:767832. doi: 10.3389/fonc.2021.767832 (PMC8674533; doi:10.3389/fonc.2021.767832)

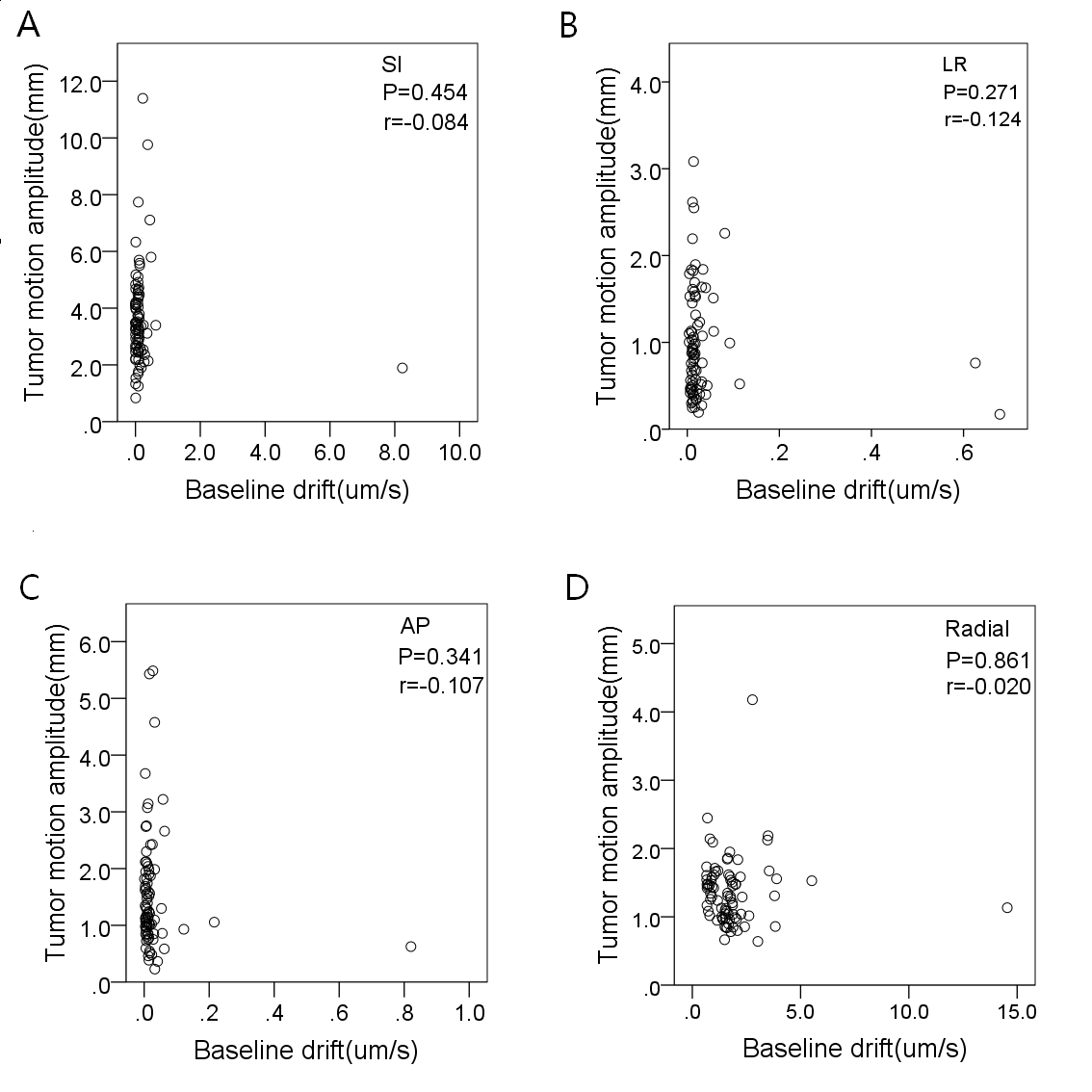

Supplement: Supplementary file 1 [file Image_1.tif]
