# Supplementary material for: Study on Motion Management of Pancreatic Cancer Treated by CyberKnife
Source: Front Oncol. 2021 Dec 2;11:767832. doi: 10.3389/fonc.2021.767832 (PMC8674533; doi:10.3389/fonc.2021.767832)
Supplement: Supplementary file 2 [file Table_1.docx]

Supplementary table 1 Treatment characteristics of 42 patients

| Characteristics | All patients (n = 42) |
| --- | --- |
| Tumor location  Pancreatic head | 10 (23.8%) |
| Pancreatic neck | 10 (23.8%) |
| Pancreatic body | 10 (23.8%) |
| Pancreatic tail | 12 (28.6%) |
| Gender  Male | 24 (57%) |
| Female | 18 (43%) |
| Age  Mean  Range | 66  41-89 |
| Number of fraction  3 fractions | 11 (26.2%) |
| 4 fractions | 17 (40.5%) |
| 5 fractions | 10 (23.8%) |
| Other | 4 (9.5%) |
| Treatment time (min) |  |
| Mean treatment time per fraction  Range | 45.9  (11.2-75.1) |

Supplementary table 2 Tracking tumor-related parameters for 42 patients

| Parameters | Mean | SD | Range |
| --- | --- | --- | --- |
| Tumor motion amplitude (mm) |  | | |
| SI | 3.66 | 1.71 | 0.83-11.39 |
| LR | 0.97 | 0.62 | 0.17-3.08 |
| AP | 1.52 | 1.02 | 0.22-5.48 |
| Radial | 1.36 | 0.49 | 0.63-4.17 |
| Respiratory motion amplitude (mm) |  | | |
| SI | 21.49 | 17.05 | 0.49-62.69 |
| LR | 5.01 | 4.99 | 0.47-21.62 |
| AP | 6.18 | 9.57 | 0.45-42.24 |
| Radial | 7.62 | 2.43 | 2.66-14.24 |
| Baseline drift (um/s) |  | | |
| SI | 20.4 | 91.1 | 0.1-823.6 |
| LR | 3.5 | 10.05 | 0. 4-67.9 |
| AP | 2.9 | 9.35 | 0.1-82.1 |
| Radial | 184. 3 | 168.8 | 66.0-1454.5 |
| Tumor volume (cm^3^) | 63.91 | 27.9 | 14.35-112.73 |
| Respiratory cycle (s) | 3.68 | 0.71 | 2.26-5.32 |

Supplementary table 3 Analysis and summary of correlation between correlation and prediction errors in 42 patients

| Parameter | Directions | Correlation error | | Prediction error | |
| --- | --- | --- | --- | --- | --- |
|  |  | r | P | r | P |
| Respiratory amplitude | SI | -0.087 | 0.441 | 0.080 | 0.479 |
|  | LR | -0.166 | 0.138 | 0.098 | 0.382 |
|  | AP | -0.123 | 0.273 | 0.418 | 0.000 |
|  | Radial | -0.026 | 0.819 | 0.222 | 0.047 |
| Tumor volume | SI | -0.193 | 0.084 | 0.144 | 0.200 |
|  | LR | -0.267 | 0.016 | 0.257 | 0.020 |
|  | AP | -0.332 | 0.002 | 0.385 | 0.000 |
|  | Radial | -0.052 | 0.647 | 0.438 | 0.000 |
| Tumor motion amplitude | SI | 0.361 | 0.001 | 0.366 | 0.001 |
|  | LR | 0.693 | 0.000 | 0.367 | 0.001 |
|  | AP | 0.457 | 0.000 | 0.633 | 0.000 |
|  | Radial | 0.379 | 0.000 | 0.515 | 0.000 |
| Baseline drift | SI | -0.300 | 0.006 | 0.255 | 0.063 |
|  | LR | -0.276 | 0.013 | 0.336 | 0.002 |
|  | AP | -0.045 | 0.000 | 0.171 | 0.126 |
|  | Radial | -0.200 | 0.073 | 0.282 | 0.011 |
| Respiratory cycle | SI | -0.036 | 0.748 | -0.222 | 0.046 |
|  | LR | 0.104 | 0.357 | -0.446 | 0.000 |
|  | AP | 0.021 | 0.851 | -0.124 | 0.271 |
|  | Radial | -0.132 | 0.240 | -0.317 | 0.004 |
| Treatment time per fraction | SI | 0.469 | 0.000 | -0.162 | 0.149 |
|  | LR | 0.480 | 0.000 | -0.063 | 0.577 |
|  | AP | 0.429 | 0.000 | 0.011 | 0.921 |
|  | Radial | 0.387 | 0.000 | -0.087 | 0.438 |
| Anatomical location | SI | -0.333 | 0.002 | 0.221 | 0.047 |
|  | LR | -0.504 | 0.000 | 0.245 | 0.028 |
|  | AP | -0.374 | 0.013 | 0.066 | 0.558 |
|  | Radial | -0.187 | 0.094 | 0.08 | 0.440 |
